# Crystal structure of *N*′-[4-(di­methyl­amino)­benzyl­idene]furan-2-carbohydrazide monohydrate

**DOI:** 10.1107/S205698902000465X

**Published:** 2020-04-09

**Authors:** Rokhaya Sylla-Gueye, Ibrahima Elhadji Thiam, James Orton, Simon Coles, Mohamed Gaye

**Affiliations:** a Département de Chimie, Faculté des Sciences et Techniques, Université Cheik Anta Diop, Dakar, Senegal; b UK National Crystallography Service, School of Chemistry, Faculty of Engineering and Physical Sciences, University of Southampton, Southampton, UK

**Keywords:** crystal structure, hydrazide, hydrazone, furoic acid

## Abstract

The condensation of 2-furoic hydrazide and 4-dimethyl amino­benzaldehyde in ethanol yielded a yellow solid formulated as the title compound, C_14_H_15_N_3_O_2_·H_2_O. The crystal packing is stabilized by inter­molecular O(water)—H⋯O,N(carbohydrazide) and N—H⋯O(water) hydrogen bonds, which form a two-dimensional network along the *bc* plane. Additional C—H⋯O inter­actions link the mol­ecules into a three-dimensional network. The dihedral angle between the mean planes of the benzene and the furan ring is 34.47 (6)°. The carbohydrazide moiety, *i.e.*, the C=N—N—C=O fragment and the benzene ring are almost coplanar, with an angle of 6.75 (9)° between their mean planes.

## Abstract 2

In the title carbohydrazide derivative, the carbohydrazide moiety is almost coplanar with the phenyl ring. The furan ring makes an angle with the phenyl ring of 34.47 (6)°. Hydrogen bonds link the mol­ecules into a two-dimensional network, which develops parallel to *bc* plane.

## Chemical context   

Furan is a colorless toxic chemical produced in various food items during heat processing and in some industrial processes (Delatour *et al.*, 2020 ▸; Rehman *et al.*, 2019 ▸; Morehouse *et al.*, 2018 ▸; Sirot *et al.*, 2019 ▸). It has been reported that furan can induce oxidative stress, endocrine disruption and toxic effects on the reproductive system of male rats (Rehman *et al.*, 2019 ▸). However, other studies have shown its ability to inhibit tyrosinase, which is an enzyme responsible for many skin disorders and diseases (Barros *et al.*, 2019 ▸). Furan derivatives, such as hydrazides, are precursors for a large variety of compounds. For example, receptors for carboxyl­ates were prepared from furoic acid hydrazide (de la Torre *et al.*, 1997 ▸). The biological activities of various furoic acid hydrazones have been evaluated against *Mycobacterium tuberculosis* (Sriram *et al.*, 2010 ▸), myelogenous leukemia cells (Silva *et al.*, 2014 ▸) and for tyrosinase inhibition (Dige *et al.*, 2019 ▸). Hydrazones of this type have also been used in the study of inter­actions of DNA with small organic or metal–organic mol­ecules to help the development of new drugs. Indeed, the elucidation of the mechanisms involved in the inter­action of DNA with these small mol­ecules makes it possible to develop models (Sathyadevi *et al.*, 2012 ▸; Sennappan *et al.*, 2019 ▸). In this paper, we report the synthesis and the characterization of the title compound, obtained from the condensation reaction between furoic acid hydrazide and 4-amino­benzaldehyde.

## Structural commentary   

**Figure 1:**
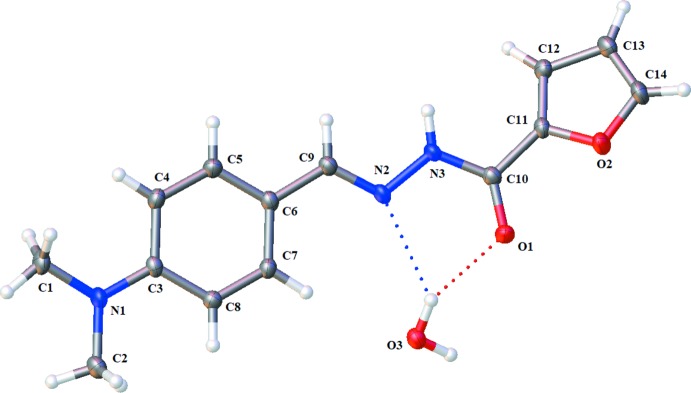
An *ORTEP* view of the title compound, showing the atom-numbering scheme and intra­molecular contacts. Displacement ellipsoids are plotted at the 50% probability level.

The mol­ecular structure of the title compound (**I**) with the atomic-labeling scheme is shown in Fig. 1 ▸. The asymmetric unit of **I** contains one mol­ecule of the Schiff base ligand and one water mol­ecule. The mol­ecule adopts an *E* configuration with respect to the C9=N2 bond. The carbohydrazide moiety, C9=N2—N3—C10=O, is almost coplanar with the benzene ring, with an angle of 6.75 (9)° between their mean planes. The C10=O1 bond length [1.2392 (16) Å], which has double-bond character, shows that the compound did not undergo enolization as observed in some furoic hydrazide derivatives (Rodríguez-Argüelles *et al.*, 2009 ▸). It exists only in the keto form. This form of the Schiff base is further confirmed by the N3—C10 [1.3383 (17) Å] and N2—N3 [1.3846 (14) Å] bond distances, which indicate that these are single bonds and by N2=C9 [1.2832 (17) Å], which is a double bond.
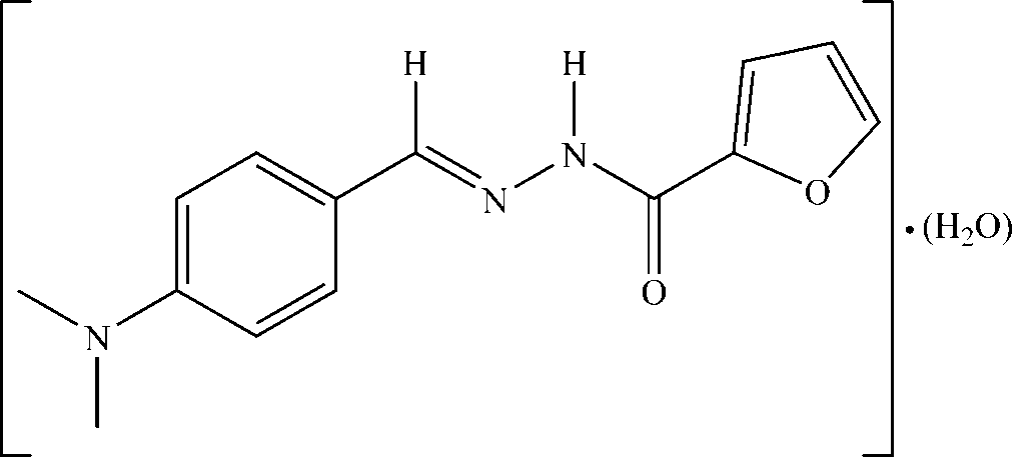


The O1 and N2 atoms are in a *syn* conformation with respect to the C10—N3 link [O1—C10—N3—N2 = −1.2 (2)°]. The dihedral angle between the benzene and the furan rings is 34.47 (6)°. The presence of the lattice water mol­ecule differentiates the title compound **I** from that reported by Li & Meng (2010 ▸). In our compound, the oxygen atom of the furan ring and the oxygen atom of the carbonyl group are in a *syn* orientation with respect to the C10—C11 bond [O1—C10—C11—O2 = −26.44 (19)°], similar to what was observed for the compound (*E*)-*N* ^’^-(2-hy­droxy­benzyl­idene)furan-2-carbohydrazide by Bikas *et al.* (2010 ▸). This is in contrast with most hydrazones from furan-2-carbohydrazide, including the anhydrous form of the title compound, which assume an *anti* conformation with respect to the link between the carbonyl atom and the C_*ipso*_ atom of the furan ring (Jiang, 2010 ▸; Li & Jian, 2010*a* ▸,*b* ▸,*c* ▸; Li & Meng, 2010 ▸).

## Supra­molecular features   

**Figure 2:**
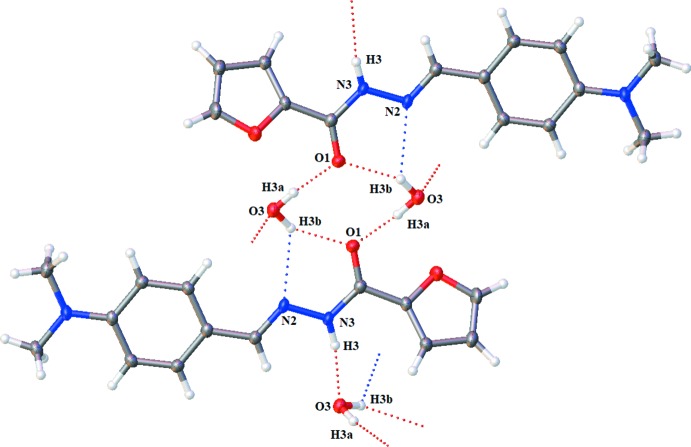
Rings and connections formed by O—H⋯O, O—H⋯N and N—H⋯O hydrogen bonds (dashed lines).

**Figure 3:**
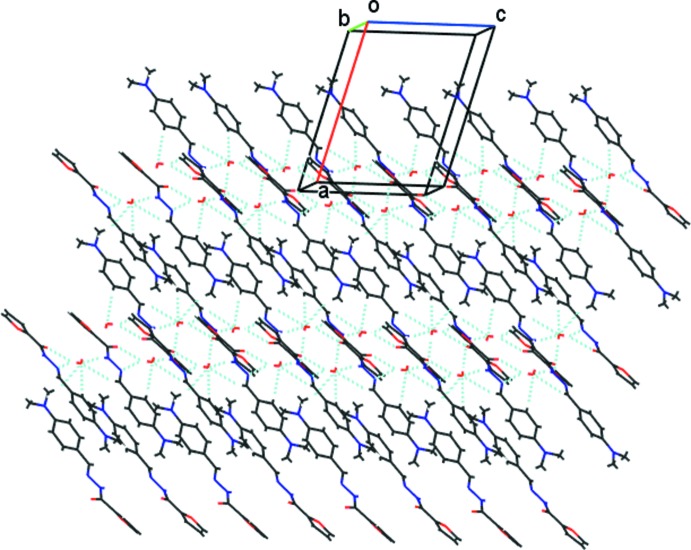
Crystal packing of the title compound, viewed along the *b* axis.

In the crystal, each independent water mol­ecule donates hydrogen bonds to the carbonyl oxygen atom of two ligand mol­ecules, forming a tetra­mer with 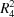
(8) rings (Fig. 2 ▸, Table 1 ▸). One of the hydrogen bonds donated by water is bifurcated between two acceptors, O1 and N2. The structure is built up further around the water mol­ecules by N—H⋯O_water_ hydrogen bonds, thus producing layers parallel to the *bc* plane. Additional C—H⋯O inter­actions inter­connect the layers and consolidate the structure into a three-dimensional network (Fig. 3 ▸).

## Database survey   

Reflecting the inter­est in compounds similar to **I**, no fewer than 43 associated structures are included in the Cambridge Structural Database (CSD version 5.40, last update November 2018; Groom *et al.*, 2016 ▸). Of these, KABNOS (Li & Meng, 2010 ▸) has the most similar structure to the title compound, the only differences being the presence of the water mol­ecule and the rotation of the furan ring around the link between the carbonyl C atom and the C_*ipso*_ atom of the furan ring in the title compound (see *Structural commentary*). Several hydrazones hits are found with the fragment furan-2-carbohydrazide. The difference between them is the substitution of the aromatic ring by a variety of groups, such as NO_2_ for AZILOM (Wang & Tai, 2016 ▸), hydroxyl for CEDZIX (Mohanraj *et al.*, 2016 ▸) and DUSZEX (Bikas *et al.*, 2010 ▸), a CH_3_ group for DUTJOS (Li & Jian, 2010*b* ▸), a meth­oxy group for EMOMUP (Cui *et al.*, 2010 ▸) or a halogen atom for GAQKEQ (Bikas *et al.*, 2012 ▸). These kinds of Schiff bases were used for preparing complexes with transition-metal or lanthanide ions. The ligand acts in a bidentate or tridentate fashion, as reported in the literature [ABUKIU (Singh *et al.*, 2017 ▸), DAZMEX (Haba *et al.*, 2005 ▸), FIGMEO (Maurya *et al.*, 2005 ▸), and VIVGOY (Alagesan *et al.*, 2014 ▸)]. One organometallic palladium complex was found containing a metal–carbon bond in a six-membered ring (TAPXEQ; Qian *et al.*, 2017 ▸). One hit corresponds to a calcium complex, in which only the carbonyl oxygen atom is coordinated to the calcium ion (YEDCIW; Tai & Wang, 2017 ▸).

## Synthesis and crystallization   

All purchased chemicals and solvents were of reagent grade and were used without further purification. The melting point was determined with a Büchi 570 melting-point apparatus and is uncorrected. To a mixture of 0.5 g (3.96 mmol) of 2-furoic hydrazide and 25 ml of ethanol were added a few drops of glacial acetic acid. A solution of 0.59 g (3.96 mmol) of 4-dimethyl amino­benzaldehyde in 25 ml of ethanol was added dropwise. The resulting mixture was stirred at 323 K for 24 h. On cooling in an ice bath, a yellow solid appeared after a few minutes. The compound was filtered off, washed with water and diethyl ether, and dried at room temperature; 0.42 g of solid was obtained (yield: 37.96%). A small qu­antity was purified by recrystallization from a di­methyl­formamide solution and yellow single crystals suitable for XRD grew within a few weeks.

## Refinement   

Crystal data, data collection and structure refinement details are summarized in Table 2 ▸. All H atoms of the ligand were located by HFIX, positioned geometrically and allowed to ride on their respective parent atoms, with C—H = 0.95 Å (C_ar_H), 0.98 Å (CH_3_) or 0.88 Å (NH). Both H atoms of the water mol­ecule were located in a difference-Fourier map, positioned geometrically and refined as a free rotating group with idealized geometry.

## Supplementary Material

Crystal structure: contains datablock(s) I. DOI: 10.1107/S205698902000465X/fy2143sup1.cif

Structure factors: contains datablock(s) I. DOI: 10.1107/S205698902000465X/fy2143Isup2.hkl

Supporting information file. DOI: 10.1107/S205698902000465X/fy2143Isup3.cml

CCDC reference: 1994610

Additional supporting information:  crystallographic information; 3D view; checkCIF report

## Figures and Tables

**Table 1:** Hydrogen-bond geometry (Å, °)

*D*—H⋯*A*	*D*—H	H⋯*A*	*D*⋯*A*	*D*—H⋯*A*
O3—H3*A*⋯O1^i^	0.87	1.92	2.7844 (15)	170
O3—H3*B*⋯O1	0.87	2.12	2.9033 (12)	150
O3—H3*B*⋯N2	0.87	2.48	3.1681 (14)	137
N3—H3⋯O3^ii^	0.88	1.95	2.7996 (14)	162
C9—H9⋯O3^ii^	0.95	2.59	3.3724 (15)	140
C12—H12⋯O1^iii^	0.95	2.43	3.3687 (16)	170
C7—H7⋯O3	0.95	2.71	3.6295 (16)	164
C1—H1*A*⋯O3^iv^	0.98	2.55	3.4057 (17)	146

Symmetry codes: (i) 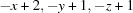; (ii) 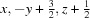; (iii) 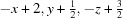; (iv) 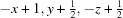.

**Table 2:** Experimental details

Crystal data	
Chemical formula	C_14_H_15_N_3_O_2_·H_2_O
*M*_r_	275.30
Crystal system, space group	Monoclinic, *P*2_1_/*c*
Temperature (K)	100
*a*, *b*, *c* (Å)	12.9328 (5), 11.2551 (4), 9.8092 (3)
β (°)	106.245 (4)
*V* (Å^3^)	1370.82 (9)
*Z*	4
Radiation type	Mo *K*α
μ (mm^−1^)	0.10
Crystal size (mm)	0.20 × 0.06 × 0.06

Data collection	
Diffractometer	XtaLAB AFC12 (RCD3)
Absorption correction	Gaussian (*CrysAlis PRO*; Rigaku OD, 2019 ▸)
*T*_min_, *T*_max_	0.536, 1.000
No. of measured, independent and observed [*I* > 2σ(*I*)] reflections	14982, 3102, 2649
*R*_int_	0.059
(sin θ/λ)_max_ (Å^−1^)	0.649

Refinement	
*R*[*F* ^2^ > 2σ(*F* ^2^)], *wR*(*F* ^2^), *S*	0.048, 0.136, 1.06
No. of reflections	3102
No. of parameters	186
H-atom treatment	H-atom parameters constrained
Δρ_max_, Δρ_min_ (e Å^−3^)	0.37, −0.35

Computer programs: *CrysAlis PRO* (Rigaku OD, 2019 ▸), *SHELXT* (Sheldrick, 2015*a* ▸), *SHELXL* (Sheldrick, 2015*b* ▸) and *OLEX2* (Dolomanov *et al.*, 2009 ▸).

## supplementary crystallographic information

### Crystal data

**Table:**

C_14_H_15_N_3_O_2_·H_2_O	*F*(000) = 584
*M**_r_* = 275.30	*D*_x_ = 1.334 Mg m^−^^3^
Monoclinic, *P*2_1_/*c*	Mo *K*α radiation, λ = 0.71075 Å
*a* = 12.9328 (5) Å	Cell parameters from 7776 reflections
*b* = 11.2551 (4) Å	θ = 2.4–31.3°
*c* = 9.8092 (3) Å	µ = 0.10 mm^−^^1^
β = 106.245 (4)°	*T* = 100 K
*V* = 1370.82 (9) Å^3^	Block, yellow
*Z* = 4	0.20 × 0.06 × 0.06 mm

### Data collection

**Table:**

XtaLAB AFC12 (RCD3) diffractometer	3102 independent reflections
Radiation source: Rotating-anode X-ray tube, Rigaku (Mo) X-ray Source	2649 reflections with *I* > 2σ(*I*)
Mirror monochromator	*R*_int_ = 0.059
Detector resolution: 10.0000 pixels mm^-1^	θ_max_ = 27.5°, θ_min_ = 2.4°
ω scans	*h* = −16→15
Absorption correction: gaussian (CrysAlis Pro; Rigaku OD, 2019)	*k* = −12→14
*T*_min_ = 0.536, *T*_max_ = 1.000	*l* = −12→12
14982 measured reflections	

### Refinement

**Table:**

Refinement on *F*^2^	Primary atom site location: dual
Least-squares matrix: full	Hydrogen site location: mixed
*R*[*F*^2^ > 2σ(*F*^2^)] = 0.048	H-atom parameters constrained
*wR*(*F*^2^) = 0.136	*w* = 1/[σ^2^(*F*_o_^2^) + (0.0786*P*)^2^ + 0.3872*P*] where *P* = (*F*_o_^2^ + 2*F*_c_^2^)/3
*S* = 1.06	(Δ/σ)_max_ = 0.001
3102 reflections	Δρ_max_ = 0.37 e Å^−^^3^
186 parameters	Δρ_min_ = −0.35 e Å^−^^3^
0 restraints	

### Special details

**Table:**

Geometry. All esds (except the esd in the dihedral angle between two l.s. planes) are estimated using the full covariance matrix. The cell esds are taken into account individually in the estimation of esds in distances, angles and torsion angles; correlations between esds in cell parameters are only used when they are defined by crystal symmetry. An approximate (isotropic) treatment of cell esds is used for estimating esds involving l.s. planes.

### Fractional atomic coordinates and isotropic or equivalent isotropic displacement parameters (Å^2^)

**Table:**

	*x*	*y*	*z*	*U*_iso_*/*U*_eq_	
O1	0.94791 (8)	0.57248 (8)	0.63770 (9)	0.0190 (2)	
O3	0.86017 (8)	0.55049 (8)	0.33216 (9)	0.0176 (2)	
H3A	0.915716	0.504655	0.340015	0.026*	
H3B	0.867014	0.575836	0.417953	0.026*	
O2	1.06257 (9)	0.57949 (9)	0.92208 (10)	0.0229 (3)	
N2	0.81295 (9)	0.75421 (10)	0.52944 (11)	0.0157 (3)	
N3	0.88775 (9)	0.75945 (10)	0.66155 (11)	0.0159 (3)	
H3	0.893005	0.823175	0.715043	0.019*	
N1	0.39936 (10)	0.87858 (10)	0.00561 (12)	0.0199 (3)	
C9	0.74829 (11)	0.84311 (11)	0.50238 (13)	0.0159 (3)	
H9	0.758000	0.905931	0.569470	0.019*	
C11	1.02928 (11)	0.68189 (11)	0.84694 (13)	0.0154 (3)	
C12	1.07767 (11)	0.77766 (12)	0.92134 (14)	0.0178 (3)	
H12	1.068840	0.858404	0.892168	0.021*	
C6	0.66077 (11)	0.85061 (11)	0.37235 (13)	0.0151 (3)	
C3	0.48293 (11)	0.86842 (11)	0.12594 (13)	0.0148 (3)	
C7	0.63960 (11)	0.76157 (11)	0.26809 (13)	0.0153 (3)	
H7	0.685817	0.694446	0.280113	0.018*	
C10	0.95190 (11)	0.66568 (11)	0.70625 (13)	0.0152 (3)	
C8	0.55339 (11)	0.76949 (11)	0.14894 (13)	0.0153 (3)	
H8	0.540804	0.707402	0.080663	0.018*	
C4	0.50366 (11)	0.95729 (12)	0.23173 (13)	0.0173 (3)	
H4	0.457358	1.024256	0.220967	0.021*	
C5	0.59054 (11)	0.94761 (11)	0.35049 (13)	0.0174 (3)	
H5	0.603117	1.008900	0.419786	0.021*	
C13	1.14452 (12)	0.73294 (13)	1.05223 (14)	0.0213 (3)	
H13	1.189178	0.778064	1.127900	0.026*	
C1	0.32241 (11)	0.97532 (13)	−0.00914 (14)	0.0213 (3)	
H1A	0.290556	0.973484	0.070497	0.032*	
H1B	0.265496	0.966333	−0.098568	0.032*	
H1C	0.359355	1.051331	−0.009268	0.032*	
C14	1.13200 (12)	0.61440 (13)	1.04763 (14)	0.0240 (3)	
H14	1.166921	0.561822	1.122047	0.029*	
C2	0.37893 (12)	0.78798 (13)	−0.10364 (14)	0.0227 (3)	
H2A	0.446986	0.764697	−0.121504	0.034*	
H2B	0.329883	0.819423	−0.191193	0.034*	
H2C	0.346096	0.718529	−0.072104	0.034*	

### Atomic displacement parameters (Å^2^)

**Table:**

	*U*^11^	*U*^22^	*U*^33^	*U*^12^	*U*^13^	*U*^23^
O1	0.0221 (6)	0.0142 (5)	0.0170 (5)	0.0019 (4)	−0.0008 (4)	−0.0029 (3)
O3	0.0184 (5)	0.0183 (5)	0.0142 (4)	0.0004 (4)	0.0014 (4)	0.0006 (3)
O2	0.0303 (6)	0.0168 (5)	0.0160 (5)	0.0017 (4)	−0.0030 (4)	0.0030 (3)
N2	0.0145 (6)	0.0170 (5)	0.0120 (5)	−0.0006 (4)	−0.0022 (4)	−0.0007 (4)
N3	0.0162 (6)	0.0155 (5)	0.0122 (5)	0.0013 (4)	−0.0022 (4)	−0.0032 (4)
N1	0.0179 (6)	0.0215 (6)	0.0159 (5)	0.0059 (4)	−0.0023 (4)	−0.0005 (4)
C9	0.0165 (7)	0.0154 (6)	0.0142 (6)	−0.0014 (5)	0.0015 (5)	−0.0011 (4)
C11	0.0145 (7)	0.0169 (6)	0.0136 (6)	0.0032 (5)	0.0016 (5)	0.0022 (4)
C12	0.0169 (7)	0.0157 (6)	0.0179 (6)	0.0029 (5)	0.0002 (5)	−0.0004 (5)
C6	0.0151 (7)	0.0150 (6)	0.0136 (6)	−0.0006 (5)	0.0016 (5)	0.0019 (4)
C3	0.0134 (7)	0.0169 (6)	0.0131 (6)	−0.0002 (5)	0.0023 (5)	0.0028 (4)
C7	0.0161 (7)	0.0135 (6)	0.0154 (6)	0.0017 (5)	0.0029 (5)	0.0024 (4)
C10	0.0153 (7)	0.0155 (6)	0.0137 (6)	−0.0014 (5)	0.0023 (5)	−0.0004 (4)
C8	0.0169 (7)	0.0144 (6)	0.0138 (6)	0.0004 (5)	0.0031 (5)	−0.0011 (4)
C4	0.0176 (7)	0.0145 (6)	0.0187 (6)	0.0037 (5)	0.0031 (5)	0.0023 (5)
C5	0.0196 (7)	0.0140 (6)	0.0167 (6)	0.0004 (5)	0.0018 (5)	−0.0019 (4)
C13	0.0187 (7)	0.0247 (7)	0.0167 (6)	0.0029 (5)	−0.0017 (5)	−0.0025 (5)
C1	0.0168 (7)	0.0230 (7)	0.0213 (7)	0.0059 (5)	0.0009 (5)	0.0033 (5)
C14	0.0274 (8)	0.0249 (7)	0.0143 (6)	0.0045 (6)	−0.0028 (5)	0.0025 (5)
C2	0.0210 (8)	0.0241 (7)	0.0181 (6)	0.0018 (5)	−0.0026 (5)	−0.0015 (5)

### Geometric parameters (Å, º)

**Table:**

O1—C10	1.2392 (16)	C6—C5	1.3979 (18)
O3—H3A	0.8701	C3—C8	1.4163 (18)
O3—H3B	0.8694	C3—C4	1.4120 (18)
O2—C11	1.3709 (15)	C7—H7	0.9500
O2—C14	1.3633 (16)	C7—C8	1.3740 (17)
N2—N3	1.3846 (14)	C8—H8	0.9500
N2—C9	1.2832 (17)	C4—H4	0.9500
N3—H3	0.8800	C4—C5	1.3780 (18)
N3—C10	1.3383 (17)	C5—H5	0.9500
N1—C3	1.3641 (16)	C13—H13	0.9500
N1—C1	1.4547 (17)	C13—C14	1.343 (2)
N1—C2	1.4491 (17)	C1—H1A	0.9800
C9—H9	0.9500	C1—H1B	0.9800
C9—C6	1.4517 (17)	C1—H1C	0.9800
C11—C12	1.3530 (18)	C14—H14	0.9500
C11—C10	1.4720 (17)	C2—H2A	0.9800
C12—H12	0.9500	C2—H2B	0.9800
C12—C13	1.4242 (18)	C2—H2C	0.9800
C6—C7	1.4030 (18)		
			
H3A—O3—H3B	104.5	N3—C10—C11	113.95 (11)
C14—O2—C11	105.77 (10)	C3—C8—H8	119.4
C9—N2—N3	113.86 (10)	C7—C8—C3	121.29 (11)
N2—N3—H3	120.7	C7—C8—H8	119.4
C10—N3—N2	118.67 (10)	C3—C4—H4	119.7
C10—N3—H3	120.7	C5—C4—C3	120.50 (12)
C3—N1—C1	120.22 (11)	C5—C4—H4	119.7
C3—N1—C2	121.15 (11)	C6—C5—H5	118.9
C2—N1—C1	118.37 (11)	C4—C5—C6	122.18 (12)
N2—C9—H9	119.0	C4—C5—H5	118.9
N2—C9—C6	121.99 (11)	C12—C13—H13	126.8
C6—C9—H9	119.0	C14—C13—C12	106.48 (12)
O2—C11—C10	115.39 (11)	C14—C13—H13	126.8
C12—C11—O2	110.58 (11)	N1—C1—H1A	109.5
C12—C11—C10	134.03 (11)	N1—C1—H1B	109.5
C11—C12—H12	127.0	N1—C1—H1C	109.5
C11—C12—C13	106.10 (12)	H1A—C1—H1B	109.5
C13—C12—H12	127.0	H1A—C1—H1C	109.5
C7—C6—C9	122.97 (12)	H1B—C1—H1C	109.5
C5—C6—C9	119.58 (11)	O2—C14—H14	124.5
C5—C6—C7	117.39 (12)	C13—C14—O2	111.06 (12)
N1—C3—C8	121.51 (11)	C13—C14—H14	124.5
N1—C3—C4	121.20 (12)	N1—C2—H2A	109.5
C4—C3—C8	117.28 (12)	N1—C2—H2B	109.5
C6—C7—H7	119.3	N1—C2—H2C	109.5
C8—C7—C6	121.34 (12)	H2A—C2—H2B	109.5
C8—C7—H7	119.3	H2A—C2—H2C	109.5
O1—C10—N3	124.19 (12)	H2B—C2—H2C	109.5
O1—C10—C11	121.86 (11)		
			
O2—C11—C12—C13	−0.78 (16)	C12—C11—C10—N3	−27.5 (2)
O2—C11—C10—O1	−26.44 (19)	C12—C13—C14—O2	0.68 (18)
O2—C11—C10—N3	153.34 (12)	C6—C7—C8—C3	0.6 (2)
N2—N3—C10—O1	−1.2 (2)	C3—C4—C5—C6	−0.5 (2)
N2—N3—C10—C11	179.07 (11)	C7—C6—C5—C4	−0.2 (2)
N2—C9—C6—C7	0.7 (2)	C10—C11—C12—C13	179.99 (15)
N2—C9—C6—C5	177.86 (13)	C8—C3—C4—C5	1.20 (19)
N3—N2—C9—C6	−176.75 (11)	C4—C3—C8—C7	−1.24 (19)
N1—C3—C8—C7	177.79 (12)	C5—C6—C7—C8	0.16 (19)
N1—C3—C4—C5	−177.83 (12)	C1—N1—C3—C8	174.21 (12)
C9—N2—N3—C10	173.35 (12)	C1—N1—C3—C4	−6.80 (19)
C9—C6—C7—C8	177.35 (12)	C14—O2—C11—C12	1.18 (16)
C9—C6—C5—C4	−177.48 (12)	C14—O2—C11—C10	−179.43 (12)
C11—O2—C14—C13	−1.14 (17)	C2—N1—C3—C8	0.3 (2)
C11—C12—C13—C14	0.07 (17)	C2—N1—C3—C4	179.28 (13)
C12—C11—C10—O1	152.75 (15)		

### Hydrogen-bond geometry (Å, º)

**Table:**

*D*—H···*A*	*D*—H	H···*A*	*D*···*A*	*D*—H···*A*
O3—H3*A*···O1^i^	0.87	1.92	2.7844 (15)	170
O3—H3*B*···O1	0.87	2.12	2.9033 (12)	150
O3—H3*B*···N2	0.87	2.48	3.1681 (14)	137
N3—H3···O3^ii^	0.88	1.95	2.7996 (14)	162
C9—H9···O3^ii^	0.95	2.59	3.3724 (15)	140
C12—H12···O1^iii^	0.95	2.43	3.3687 (16)	170
C7—H7···O3	0.95	2.71	3.6295 (16)	164
C1—H1*A*···O3^iv^	0.98	2.55	3.4057 (17)	146

Symmetry codes: (i) −*x*+2, −*y*+1, −*z*+1; (ii) *x*, −*y*+3/2, *z*+1/2; (iii) −*x*+2, *y*+1/2, −*z*+3/2; (iv) −*x*+1, *y*+1/2, −*z*+1/2.
